# Clinical, Radiographic Peri-Implant Parameters and Patient Satisfaction with Splinted and Non-splinted Short Dental Implants in the Maxillary Premolar-Molar Region: A Long-Term Retrospective Clinical Study

**DOI:** 10.3290/j.ohpd.b4347791

**Published:** 2023-08-30

**Authors:** Eman M. AlHamdan, Abdulaziz Alsahhaf, Khaled M. Alzahrani, Fahim Vohra, Tariq Abduljabbar

**Affiliations:** a Associate Professor, Department of Prosthetic Dental Sciences, College of Dentistry, King Saud University, Riyadh, Saudi Arabia. Study design, patient evaluation, data collection, analysis and interpretation, review of manuscript draft.; b Associate Professor, Department of Prosthetic Dental Sciences, College of Dentistry, King Saud University, Riyadh, Saudi Arabia. Conceptualisation of study, data interpretation, investigation, methodology, manuscript review.; c Associate Professor, Department of Prosthetic Dental sciences, College of Dentistry, Prince Sattam Bin AbdulAziz University, Alkharj, Saudi Arabia. Conceptualisation, methodology, data analysis, resources, wrote the manuscript.; d Professor, Department of Prosthetic Dental Sciences, College of Dentistry, King Saud University, Riyadh, Saudi Arabia. Conceptualisation, methodology, data analysis, resources, wrote the manuscript.; e Professor, Department of Prosthetic Dental Sciences, College of Dentistry, King Saud University, Riyadh, Saudi Arabia. Conceptualisation, study procedures, investigation, methodology, evaluations of patients, funding, validation, manuscript review.

**Keywords:** patient satisfaction, single crowns, short implants, splinted crowns

## Abstract

**Purpose:**

The goal of this study was to assess the peri-implant condition, peri-implant bone loss (PBL), and complication rates of short dental implant-supported splinted crowns (SDI-SCs) and non-splinted crowns (SDI-NSCs) in the maxillary premolar-molar region.

**Materials and Methods:**

Patients who had short implants placed near their maxillary sinuses were evaluated. Both patient satisfaction and presence of any technical complication, e.g. porcelain wear and chipping, loss of retention and loosening of the abutment, fixture or screw, were noted. The peri-implant plaque index (PIPI), probing depth (PIPD), bleeding on probing (PIBP), and peri-implant bone loss (PBL) were evaluated. To assess the impact of prosthesis type and SDI placement on technical problems, a log-rank test was computed. p < 0.05 was considered statistically significant.

**Results:**

A total of 72 patients agreed to be followed-up, showing a mean follow-up time of 3.1 years. Ninty-five implants in total (55 SDI-SCs, and 40 SDI-NSCs) with moderately rough surfaces were evaluated. The average PBL score for implant and patients was 1.27 (0.02–3.97) and 1.25 (0.03–4.41), respectively. More technical complications were observed with single crowns than with splinted crowns. There were no statistically significant differences in the peri-implant parameters between SDI-SCs and SDI-NSCs (p > 0.05). PBL at molar sites was substantially higher than at premolar sites (p = 0.048). Sixty patients (83.3%) were satisfied with the appearance of the crowns, while 57 patients (79.1%) were satisfied with the crowns’ performance.

**Conclusion:**

The peri-implant conditions, bone levels, technical complication rates and patient satisfaction were comparable between the SDI-SCs and SDI-NSCs. However, implants placed in the molar sites had statistically significantly greater bone loss in comparison to those at the premolar sites.

Dental implant treatment is today considered a universally acclaimed method for restoring partial or complete edentulous dental arches.^20,29^ This treatment restores function and aesthetics and at the same time minimises bone resorption, which is a likely outcome due to load transmission from the implants to the jaw bone.^18,28^ Factors such as competent oral hygiene, primary stability of the implant, osseointegration, and inhibition of soft tissue inflammation regulate the chances of dental implant survival.^12,25,26^ However, because stress is immediately transferred to bone tissue during functional loading, high loading in the presence of factors such as systemic disease, smoking, trauma and/or periodontitis may accelerate the rate at which bone resorption occurs.^9,29^

Recently, dental implant practitioners have been using short dental implants (SDIs) more frequently. The term ‘short dental implants (SDI)’ is subjective, with no established consensus regarding its proper definition.^7^ Some authors described SDIs as implants having a length of 10 mm, while other clinicians defined them as ‘implants no longer than 7 mm.’^11^ This type of dental implant can be used in areas having inadequate bone volume without the need of performing complex surgical interventions such as bone augmentation, distraction osteogenesis, and sinus floor elevation.^3,15,24^ In terms of posterior maxillary atrophic ridges, short dental implants (SDIs) are a good way to restore edentulous spaces in this area. Moreover, this alternative treatment strategy is advantageous for the patient, as it is relatively inexpensive and requires less operating time.^23^ Regarding its success rate, a review by Esfahrood et al^14^ found that the survival rate of short implants is high when placed in the maxillary posterior edentulous areas. Similarly, a two-year retrospective study by Renouard et al^27^ reported a 95% cumulative survival rate of short dental implants in the severely resorbed maxilla. Furthermore, SDIs used to restore partially or completely edentulous mandibles with fixed or removable prostheses showed a 99% survival rate.^16^ Thus, Grant et al^16^ concluded that SDI can be considered a good treatment alternative to complex surgical procedures to treat atrophied mandibular ridges.

One of the most popular restorations in implant dentistry is the non-splinted crown (NSC) or single crown restoration. In comparison to previous fixed partial restorations, these restorations provide a more comfortable prosthetic approach with improved emergence profiles and greater oral hygiene access.^13,20^ Splinted prostheses are a preferable owing to their better mechanical properties. Yilmaz et al^34^ suggested that splinting short implants would provide an even distribution of strain during functional loading. Similarly, Lemos et al^22^ performed a finite element analysis in which they observed better stress distribution after the prostheses were splinted.

Numerous studies exist in which standard dental implants supporting both non-splinted and splinted crowns showed positive outcomes in terms of survival, improved peri-implant conditions, and less bone loss. However, knowledge is limited when it comes to the use of short implants supporting both the single crown and splinted crowns in the posterior maxillary region. Therefore, the aim of this study was to study the peri-implant parameters, complications, patient satisfaction, and bone loss around SDIs supported with splinted (SDI-SCs) and non-splinted crowns (SDI-NSCs) placed in the maxillary premolar-molar region.

## Materials and Methods

### Ethical Clearance and Study Plan

The present retrospective study was conducted at the Centre for Specialist Dental Practice, Riyadh, Saudi Arabia, in adherence to the ethical principles proposed in the Declaration of Helsinki.^17^ The study protocol was reviewed by the Ethics and Research Committee of the Centre for Specialist Dental Practice and Clinical Research, Saudi Arabia (UDCRC-RB-12-22). The eligible candidates were invited for a follow-up examination via e-mails and phone calls. Upon arrival, the participants were asked to read and fill out a consent sheet which comprised information regarding the aims and objectives of the research. Each patient was free to leave the study at any time without facing any consequences.

### Patient Selection

Individuals aged ≥30 years and having short implants with SDI-SCs and SDI-NSCs placed in the area of the maxillary sinus were selected. The exclusion criteria included habitual smokers; diagnosis of uncontrolled diabetes mellitus; compromised periodontal health; history of any periodontal surgery (e.g. bone augmentation); and complete edentulism.

### Assessment of Implants and Prostheses

Implants were inserted in one-stage surgery only if bone densitometry was >400 Hounsfield units and the initial stability ranged between 45 and 60 N. One single American Board-certified, surgically-trained prosthodontist (T.A.) performed the clinical procedures. The recordings included baseline demographics (age, gender) and implant and prosthetic-related assessments. These included the number of implants, implant length, implant location, type of prosthetic restoration (splinted crowns [SC] or non-splinted crowns [NSCs]), and complications (i.e. porcelain wear, loss of retention and loosening of the abutment, fixture or screw).^21^ In this study, the platform-switched short implants with dimensions of 4.6 mm diameter and 6.0 mm length (Biohorizons; Birmingham, AL, USA) were placed in the maxillary premolar-molar region. The participants were either given SCs or NSCs based on the designed protocol; a total of 75 screw-retained (SR) and 35 cement-retained (CR) restorations were provided.

### Clinical Assessment

All clinical assessments were evaluated using the recommendations and guidelines described in the Consensus Report of the Eleventh European Workshop on Periodontology.^32^

### Peri-implant Parameters

An expert examiner (TA) recorded the values of the following peri-implant parameters: peri-implant plaque index (PIPI); peri-implant bleeding on probing (PIBP); and implant probing depth (PIPD). These parameters were checked at six sites: mesiolingual, mid-lingual, distolingual, mesiobuccal, mid-buccal and distobuccal. PIPI and PIBP were scored dichotomously: 0 = plaque/bleeding was absent; 1 = plaque/bleeding was present. PIPD was recorded by using a periodontal probe (University of North Carolina [UNC-15], Hu-Friedy; Chicago, IL, USA).

### Radiographic Assessment

Digital peri-apical radiographs were incorporated into specialised software (ROMEXIS, Planmeca; Helsinki, Finland). These images were examined on a standardised computer screen (Samsung SyncMaster digital TV monitor; Seoul, Korea) using an image analyser (Image Tool 3.0 Program; San Antonio, TX, USA).^19^ The radiographs were taken as described in previous studies.^2,6^ Peri-implant bone loss was (PIBL) calculated as the total vertical distance from the crest of the alveolar bone to the topmost supracrestal part of the dental implant, which was standardised from the baseline value.^1^ PIBL values were reported as means ± SD. The incorporation and analysis of the digital radiographs and measurement of PIBL were carried out by one trained examiner.

### Patient Satisfaction

A questionnaire containing items related to function and aesthetics of the restoration was filled out by all participants. The participants were required to answer sections containing the visual analogue Likert scale, ranging from ‘extremely satisfied’ to ‘extremely dissatisfied’.

### Statistical Analysis

Statistical analysis was performed using SPSS v23 (Chicago, IL, USA). Shapiro-Wilk tests were used to calculate the dependent variables’ normal distribution. One-way ANOVA was used to examine the significance of comparisons between groups of means for all clinical indicators and PBL. For multiple comparisons, Bonferroni’s post-hoc adjustment was performed. The rate of complications was estimated both at the patient (statistical unit) and the implant (statistical unit) level. To assess the impact of prosthesis type and short dental implant location on technical problems, the log-rank test was conducted. p-values < 0.05 were considered statistically significant.

## Results

Table 1 presents the demographic data of the participating individuals. Eighty (80) patients who met the requirements for inclusion received phone calls inviting them to participate in the study. Seventy-two (72) of them (50 men and 22 women) consented to follow-up and were enrolled in the study.

**Table 1:** Patient demographics

Characteristics	Findings
Number of participants (n)	72
Gender (F/M)	22/50
Mean follow-up (years)	3.1 ± 0.2

F: female; M: male.

A total of ninety-five (95) platform-switched (PS) short dental implants (SDI) were included in the present study. Out of these, 40 SDI-NSCs and 55 SDI-SCs were placed in the premolar-molar region in the maxillary bone. All implants studied were 6 mm in length and 4.6 mm in diameter, and were placed at the bone level. A total of sixty-three (63) screw-retained and thirty-two (32) cement-retained prosthetic restorations were placed on the implants (Table 2).

**Table 2:** Implant-related parameters

Peri-implant parameters	Short dental implant-supported non-splinted crowns (SDI-NSCs)	Short dental implant-supported splinted crowns (SDI-SCs)
Total implants placed	40	55
**Location of implant placement**		
Maxillary premolar area Maxillary molar area	24 16	35 20
Depth of implant placement	Bone level	Bone level
Design of implant	PS	PS
Implant length and diameter (mm)	6, 4.6	6, 4.6
Implant loading after placement (months)	3.7 ± 0.4	3.2 ± 0.2
Prosthetic restoration CR, SR	12, 28	20, 35
Duration of implants in function (years)	3.2 ± 0.3	3.6 ± 0.4

PS: platform switched; CR: cement retained; SR: screw retained.

The clinical peri-implant parameters (PIPI, PIBP, and PIPD) around both the SDI-NSCs and SDI-SCs are given in Table 3. For SDI-NSCs, PIPI and PIBP were recorded as 27.5% and 16.5%, respectively. Similarly, the PIPI and PIBOP observed in SDI-SCs were 34.5% and 27.1%, respectively. The mean PIPD for SDI-NSCs and SDI-SCs was reported to be 3.6 mm and 3.4 mm at follow-up, respectively. None of the clinical peri-implant parameters including PIPI (p = 0.93), PIBP (p = 0.08) or PIPD (p = 0.54) showed any statistically significant differences between SDI-NSCs and SDI-SCs (Table 3).

**Table 3:** Peri-implant parameters of the study groups

Peri-implant parameters	Short dental implant-supported *non-splinted crowns (SDI-NSCs)	Short dental implant-supported splinted crowns (SDI-SCs)	p-value
PIPI (% in sites)	27.5 ± 3.9^a^	34.5 ± 4.1^a^	0.93
PIBP (% in sites)	26.5 ± 4.2^a^	27.1 ± 3.9^a^	0.08
PIPD (mm)	3.6 ± 0.3^a^	3.4 ± 0.5^a^	0.54

PIPI: peri-implant plaque index; PIBP: peri-implant bleeding on probing; PIPD: peri-implant pocket depth. Similar superscript letters indicate statistically insignificant difference. Values are means ± SD.

At the mesial and distal sites, the mean values of PBL at the implant level were 1.32 and 1.22 mm, respectively. At the patient level, the mean PBL values were 1.22 mm at the mesial site and 1.28 mm at the distal location. At the implant level, the average PBL score was 1.27 (0.02-3.97), whereas at the patient level, it was 1.25 (0.02-4.41). Additionally, data stratification based on PBL distribution was calculated. Thirty-nine (39) implants (or 41%) and 42 patients (or 58.3%) revealed PBL ranging from 0 to 1 mm. PBL ranged from 1 to 2 mm in 49 implants (51.5%) and 25 patients (34.7%), but ranged from 2 to 4 mm in 5 implants (5.2%) and 3 individuals (4.16%). PBL >4 mm was seen in 2 implants (2.1%) and 2 patients (2.77%) (Table 4).

**Table 4:** Bone levels at implant and patient levels

Measurements	Implant level		
	Mesial	Distal	Average
Mean (mm)	1.32	1.22	1.27
SD (% in sites)	0.05	0.12	0.085
Range (mm)	0.02-4.02	0.00-3.92	0.00-3.97
0-1 mm	40 (42.1%)	38 (40%)	39 (41.0%)
1-2 mm	47 (49.4%)	51 (53.6%)	49 (51.5%)
2-4 mm	6 (6.3%)	4 (4.2%)	5 (5.2%)
>4 mm	2 (2.1%)	2 (2.1%)	2 (2.1%)
Total	95 (100.0%)	95 (100.0%)	95 (100.0%)
Measurements	Patient level		
	Mesial	Distal	Average
Mean (mm)	1.22	1.28	1.25
SD (% in sites)	0.25	0.20	0.225
Range (mm)	0.02-4.71	0.00-4.12	0.00-4.41
0-1 mm	45 (62.5%)	39 (54.1%)	42 (58.3%)
1-2 mm	22 (30.5%)	28 (38.8%)	25 (34.7%)
2-4 mm	3 (4.16%)	3 (4.16%)	3 (4.16%)
>4 mm	2 (2.77%)	2 (2.77%)	2 (2.77%)
Total	72 (100.0%)	72 (100.0%)	72 (100.0%)

SD: standard deviation.

Table 5 shows how prosthesis type and implant site influenced technical difficulties and bone loss seen at the peri-implant level. In comparison to splinted crowns, the rates of technical complications with SDI-NSCs were statistically significantly higher (p = 0.03). However, the difference in the PBL between the two types of restorations was not statistically significant (p = 0.72). In comparison to implants placed in premolar locations, the PBL of implants placed in molar sites was statistically significantly higher (p = 0.04). The difference in the technical complications in the molar and premolar areas was statistically insignificant.

**Table 5:** Influence of prosthesis type and implant location on technical complications and peri-implant bone loss

	Technical complications	Peri-implant bone loss
Non-splinted crowns	12/40 (30%)	1.21 ± 0.02 (n = 40)
Splinted crowns	8/55 (14.5%)	1.35 ± 0.03 (n = 55)
p-value	0.03	0.72
Maxillary premolar area	20/59 (33.8%)	1.25 ± 0.03 (n = 59)
Maxillary molar area	11/36 (30.5%)	1.89 ± 0.01 (n = 36)
p-value	0.37	0.04

Table 6 presents the percentage of patient satisfaction. Sixty (60) patients (83.3%) were content with the appearance of the crowns, while 57 patients (79.1%) were satisfied with the crown’s performance.

**Table 6:** Patient satisfaction

	Satisfied patients (%)	Unsatisfied patients (%)
Aesthetic outcomes	60 (83.3%)	12 (16.6%)
Functional outcomes	57 (79.1%)	15 (20.8%)

## Discussion

The aim of the current retrospecitve study was to evaluate the bone loss, patient satisfaction, peri-implant problems, and complication rates of patients with SDI-SCs and SDI-NSCs present in the maxillary premolar-molar region. The results of the study reflected a relatively high rate of patient satisfaction. Moreover, the clinical and peri-implant parameters observed during the follow-ups showed comparable outcomes for the SDI-SCs and SDI-NSCs.

According to the results observed at follow-up, a positive trend towards increased chances of survival can be expected. This may be due to the presence of sound and improved clinical parameters during the study. Moreover, good oral hygiene was evident in all the participating individuals. This evidence of good oral hygiene reflects the continuous attention patients pay to their respective oral hygiene. In addition to this, the presence of healthy peri-implant pockets is another sign that the patients strictly followed the oral hygiene instructions given during the prosthesis placement.^28^

According to the observed results, the rates of technical complications of SDI-NSCs (30%) were statistically significantly greater than their counterparts, i.e. splinted crowns (SCs; 14.5%). Loss of retention and porcelain chipping were encountered in 12 restorations in NSCs. The incidence of retention loss was more commonly seen in the cement-retained than screw-retained restorations. Cement-retained prosthesis are difficult to remove, and due to the difficulty of removing excess cement, they are biologically weak. Cement residues in the gingiva may induce soft-tissue inflammation in the implant-gingival area and eventually cause retention loss.^28^ The results obtained from the present study correspond with the study by Assaf et al,^10^ in which SDI-NSCs were associated with more technical complications than were splinted restorations (SCs).

For peri-implant bone loss, no stastistically significant differences were observed between the SDI-NSCs and SDI-SCs at follow-up. Shi et al^30^ reported outcomes that corroborated with the results of the present study. Furthermore, the results reported in the systematic review by Al Amri et al^4^ also showed similar outcomes. The study observed no statistically significant changes in the levels of peri-implant bone with non-splinted and splinted implant crowns. In addition, in a retrospective study by Al-Sawaf et al,^7^ the PBL levels exhibited no statistically significant changes for SDI-NSCs and SDI-SCs. Since the PBL values seen at follow-up for both SDI-NSCs and SDI-SCs were not considered clinically significant, it may indicate that both types of prosthesis play a role in preserving the bone levels at the crestal margin. The average peri-implant bone loss levels associated with short dental implants placed at maxillary molar sites (1.89 mm) were statistically significantly greater than at maxillary premolar sites (1.25 mm). One of the factors that may be associated with these findings is the presence of greater occlusal loads in the maxillary molar areas in comparison to the premolars. In addition, cleaning and oral hygiene maintenance in the maxillary molar regions can also be considered one of the factors that should not be disregarded.^4^

Although the clinicians adopted a very strict policy of inclusion and exclusion, the present study has a few limitations. First, the study sample was small. A larger sample size might have enabled the authors to derive more robust clinical outcomes, including peri-implant outcomes, technical complications, and patient satisfaction. Moreover, microbiological analysis would have provided the researchers with additional knowledge on the microbial flora associated with the physiological changes encountered during the study. The two-dimensional evaluation of the peri-implant bone levels is another limitation. The peri-apical radiographic examinations could only assess bone remodeling at the mesial and distal levels.

## Conclusion

Peri-implant conditions, bone levels, technical complication rates and patient satisfaction were comparable among the SDI-SCs and SDI-NSCs. However, implants placed at the molar sites had statistically significantly greater bone loss in comparison to those at the premolar sites.
